# Comparative analysis of therapeutic effects between medium cut-off and high flux dialyzers using metabolomics and proteomics: exploratory, prospective study in hemodialysis

**DOI:** 10.1038/s41598-021-96974-5

**Published:** 2021-08-30

**Authors:** Hyo Jin Kim, Eun Young Seong, Wonho Lee, Suhkmann Kim, Hee-Sung Ahn, Jeonghun Yeom, Kyunggon Kim, Chae Hwa Kwon, Sang Heon Song

**Affiliations:** 1 Department of Internal Medicine, Pusan National University Hospital, Busan, Korea; 2 Biomedical Research Institute, Pusan National University Hospital, Busan, Korea; 3 Department of Chemistry, Center for Proteome Biophysics and Chemistry Institute for Functional Materials, Pusan National University, Busan, Korea; 4 Asan Institute for Life Sciences, Asan Medical Center, Seoul, Korea; 5 Convergence Medicine Research Center, Asan Institute for Life Sciences, Seoul, Korea; 6 Department of Biomedical Sciences, University of Ulsan College of Medicine, Ulsan, Korea

**Keywords:** Computational biology and bioinformatics, Nephrology

## Abstract

In this single-center prospective study of 20 patients receiving maintenance hemodialysis (HD), we compared the therapeutic effects of medium cut-off (MCO) and high flux (HF) dialyzers using metabolomics and proteomics. A consecutive dialyzer membrane was used for 15-week study periods: 1st HF dialyzer, MCO dialyzer, 2nd HF dialyzer, for 5 weeks respectively. ^1^H-nuclear magnetic resonance was used to identify the metabolites and liquid chromatography-tandem mass spectrometry (LC–MS/MS) analysis was used to identify proteins. To compare the effects of the HF and MCO dialyzers, orthogonal projection to latent structure discriminant analysis (OPLS-DA) was performed. OPLS-DA showed that metabolite characteristics could be significantly classified by 1st HF and MCO dialyzers. The Pre-HD metabolites with variable importance in projection scores ≥ 1.0 in both 1st HF versus MCO and MCO versus 2nd HF were succinate, glutamate, and histidine. The pre-HD levels of succinate and histidine were significantly lower, while those of glutamate were significantly higher in MCO period than in the HF period. OPLS-DA of the proteome also substantially separated 1st HF and MCO periods. Plasma pre-HD levels of fibronectin 1 were significantly higher, and those of complement component 4B and retinol-binding protein 4 were significantly lower in MCO than in the 1st HF period. Interestingly, as per Ingenuity Pathway Analysis, an increase in epithelial cell proliferation and a decrease in endothelial cell apoptosis occurred during the MCO period. Overall, our results suggest that the use of MCO dialyzers results in characteristic metabolomics and proteomics profiles during HD compared with HF dialyzers, which might be related to oxidative stress, insulin resistance, complement-coagulation axis, inflammation, and nutrition.

The number of patients receiving hemodialysis (HD) is increasing, and despite various efforts, their survival rate is low. For instance, in Korea, the number of registered HD patients was 13,943 based on laboratory data from the End-stage Renal Disease Registry of the Korean Society of Nephrology from 2007 to 2017; among these, 3,139 deaths were recorded (22.5% patients)^1^. One of the main reasons for the high mortality rate of such patients is that HD cannot remove enough uremic toxins, as normal kidneys do^2^. Furthermore, patients on HD have a higher risk of sudden cardiac death, which is associated with cardiac comorbidities^3^.

Uremic toxins are classified as small molecules (molecular weight (MW) < 500 Dalton (Da)) and middle molecules (MW 500–60,000 Da) according to MW. Recently, large middle molecules (MW 15,000–60,000 Da) were subdivided from middle molecules, as compared to conventional middle molecules (MW 500–15,000 Da), and there are ongoing efforts to remove better them during HD. Importantly, while small and conventional middle molecules can be efficiently removed using a high flux (HF) membrane, the removal of large middle molecules is limited using this approach^4^. In fact, HF dialysis does not present a clear advantage in terms of mortality over low-flux dialysis; specifically, the survival benefits of HF dialysis were only shown in patients with diabetes or hypoalbuminemia^5^. Another dialysis modality, hemodiafiltration (HDF) has higher efficiency in removing middle molecules than HD using an HF membrane because HDF can enhance the convection effect during dialysis^6^. Analyses of the survival benefits of HDF and HD using an HF membrane have shown conflicting results; however, a recent study suggested that HDF with a highly sufficient convective volume improves patient survival^7^. However, there are several factors to consider to achieve optimal HDF: large convective volume, optimal vascular access to increase the blood flow rate, and strict water quality management. In addition, HDF is not covered by medical insurance in Korea, limiting its broad application.

A medium-cut off (MCO) dialyzer with a specific pore size and relatively narrow pore distribution was recently developed to efficiently remove middle molecules without significant albumin loss^8^. In addition, MCO dialyzers are associated with increased convective transport owing to enhanced internal filtration. Because of these characteristics, MCO dialyzers are associated with a higher large middle molecule removal rate compared to that obtained with HD using an HF dialyzer or HDF^8,9^.

Metabolomics and proteomics are important “omics” research fields, along with transcriptomics and genomics, which reveal the “functional” consequences of the genome^10^. Metabolomic studies are concerned with metabolite processes, metabolic mechanisms, and important biomarkers related to metabolic features^11,12^. Proteomics comprise the analysis of the entire protein components of a cell, tissue, or organism, as well as of the protein structures, physiological roles, and functions^13^. Importantly, the metabolome and proteome reflect the influence of environmental factors, in addition to genetic coding^10^. Therefore, the circulating levels of metabolites and proteins are dynamic and modifiable, including in the context of therapeutics. For instance, dialysis-related factors, as well as uremic toxins might affect the metabolome and proteome of HD patients. A previous metabolomics study using capillary electrophoresis time-of-flight mass spectrometry showed plasma metabolite differences between patients subjected to low- and high-flux HD^14^. In another study demonstrated that the types of detected proteins were similar; however, the degree to which specific proteins were reduced differed between low- and high-flux dialyzers, based on proteomics^15^. Since the capacity of MCO and HF dialyzers to remove large middle molecules is different, we hypothesized that these dialyzers might have different effects on the metabolism and uremic toxin clearance in the clinical context. Therefore, the aim of this study was to analyze the therapeutic effects and define the characteristics of MCO dialyzers compared to those of HF dialyzers using metabolomics and proteomics.

## Results

### Demographic characteristics and laboratory parameters of the study patients during each dialyzer period

**Figure 1:**
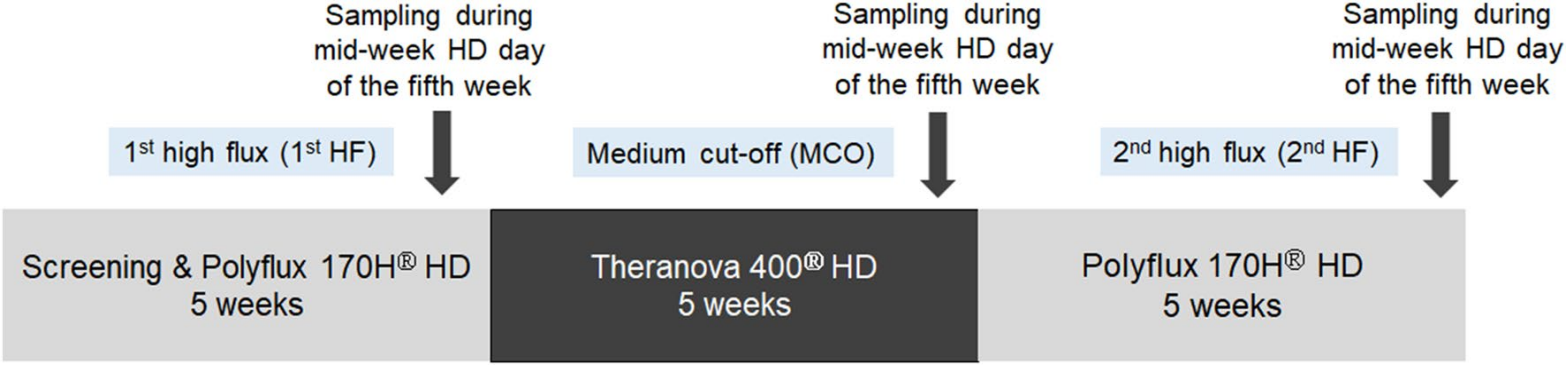
Study timeline. HD, hemodialysis; HF, high flux; MCO, medium cut-off.

The baseline characteristics of the study patients are presented in Supplemental Table 1. The mean age of patients was 59.9 ± 14.2 years and 50% were men. The mean dialysis duration was 92.1 ± 54.6 months and 80% of the patients underwent HD via native vascular access. Different dialyzer membranes were used for 3 consecutive periods of 5 weeks: 1st HF dialyzer, MCO dialyzer, and 2nd HF dialyzer, respectively (Fig. 1). The laboratory parameters of each dialyzer periods are presented in Table 1. The levels of blood urea nitrogen (*p* = 0.262), creatinine (*p* = 0.302), and single-pool Kt/V (*p* = 0.222) were not significantly different among the different dialyzer periods. Serum albumin was also similar among each dialyzer period (*p* = 0.505).

**Table 1:** Laboratory parameters during each dialyzer period.

Variables (N = 20)	1st high-flux HD (1)	MCO HD (2)	2nd high-flux HD (3)	Overall *P*	Post *hoc P*		
					(1) versus (2)	(1) versus (3)	(2) versus (3)
Hemoglobin (g/dL)	10.5 ± 1.0	10.7 ± 1.2	10.5 ± 1.1	0.562	0.312	0.984	0.423
Total protein (g/dL)	6.8 ± 0.4	6.8 ± 0.4	6.9 ± 0.3	0.395	0.701	0.326	0.159
Albumin (g/dL)	4.2 ± 0.3	4.2 ± 0.4	4.1 ± 0.3	0.505	0.716	0.245	0.453
Blood urea nitrogen (mg/dL)	56.9 ± 11.8	52.0 ± 11.4	54.4 ± 13.8	0.262	0.123	0.425	0.383
Creatinine (mg/dL)	9.5 ± 1.7	9.6 ± 1.7	9.3 ± 1.8	0.302	0.674	0.345	0.167
Corrected calcium (mg/dL)^A^	8.8 ± 0.5	9.0 ± 0.6	9.0 ± 0.7	0.211	0.021	0.197	0.831
Phosphorus (mg/dL)	5.1 ± 1.4	5.2 ± 1.2	5.3 ± 1.8	0.824	0.774	0.582	0.721
Single-pool Kt/V	1.86 ± 0.34	1.92 ± 0.35	1.85 ± 0.34	0.222	0.168	0.843	0.210

HD, hemodialysis; MCO, medium cutoff.

^A^Corrected Ca (mg/dL) = measured total Ca (mg/dL) + 0.8 × [4 − measured serum albumin (g/dL)]. *P* value obtained using repeated measures one-way analysis of variance.

### Metabolomic analysis of each dialyzer period

**Figure 2:**
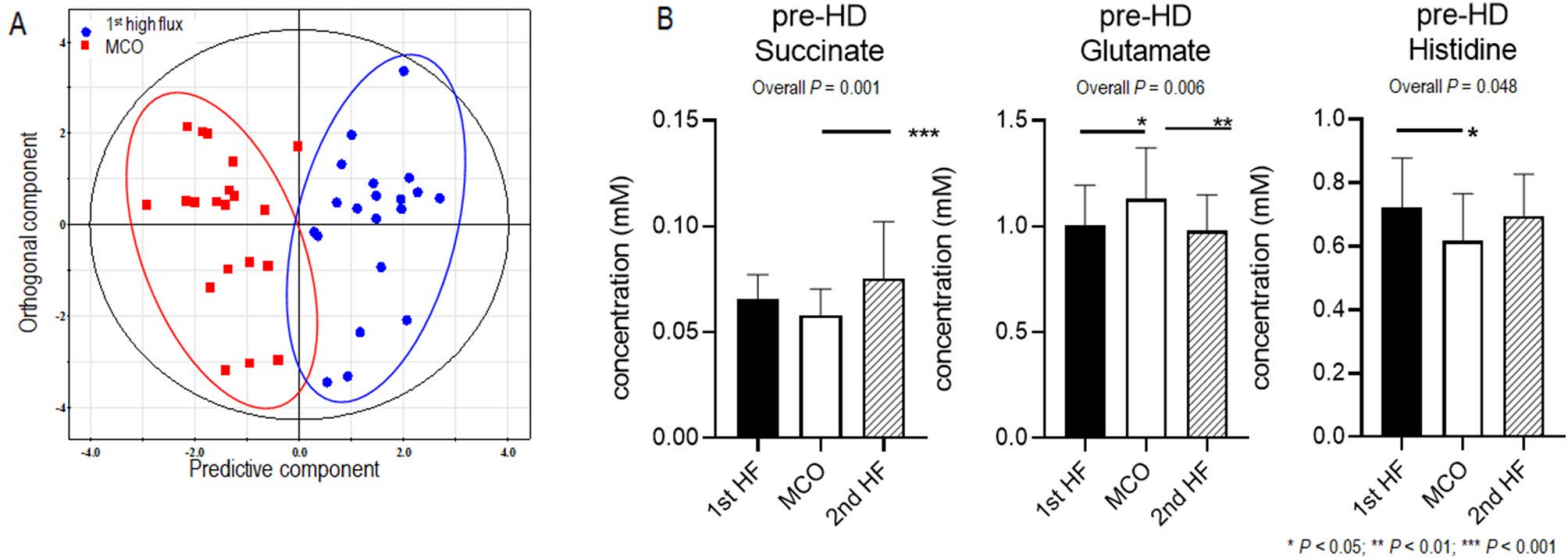
Metabolomics profiles according to each dialyzer period. (**A**) OPLS-DA score scatter plot derived from the ^1^H NMR spectra of pre-dialysis serum obtained from subjects in the 1st HF and MCO dialyzer periods. In the OPLS-DA result, the score scatter plot (R^2^ = 0.814, Q^2^ = 0.368) shows a substantial separation of the 1st HF (blue circle) and MCO (red square) dialyzer periods, suggesting significant differences between these groups. Each symbol represents the metabolomic profile of an individual sample. One predictive and one orthogonal component was used for this model. The X-axis represents the covariation and the Y-axis the corresponding orthogonal score. SIMCA-P + version 12.0 software (Umetrics, Umea, Sweden) was used to perform OPLS-DA. (**B**) Pre-HD concentration of metabolites with a VIP score > 1.0 in both the 1st HF versus MCO and MCO versus 2nd HF dialyzer comparisons. The *P* values were calculated using the repeated measures one-way analysis of variance using GraphPad Prism version 9.0.0 for Windows (GraphPad Software, San Diego, California USA, www.graphpad.com): * < 0.05, ** < 0.01, *** < 0.001. OPLS-DA, orthogonal partial least-squares discriminant analysis; NMR, nuclear magnetic resonance; HF, high flux; MCO, medium cut-off; VIP, variable important in projection; HD, hemodialysis.

The serum metabolites were evaluated in HD patients using ^1^H- nuclear magnetic resonance (NMR) spectroscopy; multivariable analysis was further performed to identify the metabolic signatures associated with HF and MCO dialyzers and determine the clinical significance of the differences detected. In total, 44 metabolites were quantified in the serum. To maximize the separation between groups, the orthogonal projection to latent structure discriminant analysis (OPLS-DA) model was applied. The score scatter plot (R^2^ = 0.814, Q^2^ = 0.368) showed that the 1st HF and the MCO dialyzers were separated substantially from each other, indicating significant differences between these groups (Fig. 2A). The variable importance in projection (VIP) score indicated the metabolites contributing to the differences between groups in the OPLS-DA model. Urea, glycerol, phenylalanine, creatine phosphate, lactate, histidine, succinate, glutamate, alanine, trimethylamine N-oxide, methionine, threonine, myo-Inositol, isobutyrate, and dimethylamine, with VIP scores ≥ 1.0, were considered significant metabolites in the separation between the 1st HF and MCO pre-HD serum samples (Table 2). The Pre-HD concentrations of metabolites with a VIP score ≥ 1.0 between the 1st HF and MCO dialyzer samples are shown in Supplemental Fig. 1. We also analyzed the VIP scores of metabolites between MCO and 2nd HF dialyzer samples. Serine, succinate, glutamate, myo-Inositol, histidine, asparagine, isoleucine, betaine, 2-phenylpropionate, and glutamine, with VIP scores > 1.0, were considered significant metabolites for the separation between MCO and 2nd HF pre-HD serum samples (Supplemental Table 2). The pre-HD concentrations of metabolites with a VIP score > 1.0 in both 1st HF versus MCO and MCO versus 2nd HF dialyzer comparisons and significantly different concentrations in the MCO period compared to the HF period are depicted in Fig. 2B. Pre-HD succinate (*p* = 0.001) and histidine (*p* = 0.048) were significantly decreased and those of glutamate (*p* = 0.006) were significantly increased in the MCO dialyzer period. The succinate concentration tended to decrease during the MCO period compared to the 1st HF period; however it increased significantly during the 2nd HF period. Similarly, the histidine levels decreased significantly during the MCO period compared to the 1st HF period and tended to increase again during the 2nd HF period. On the other hand, the glutamate levels increased significantly during the MCO period compared to the 1st HF period and then decreased significantly during the 2nd HF period.

**Table 2:** VIP score of metabolites differentially expressed between the 1st HF and MCO dialyzer samples.

Metabolites	VIP score
Urea	2.64669
Glycerol	1.67161
Phenylalanine	1.61468
Creatinine phosphate	1.56662
Lactate	1.52308
Histidine	1.52238
Succinate	1.46111
Glutamate	1.25617
Alanine	1.10633
Trimethylamine N-oxide	1.10160
Methionine	1.09604
Threonine	1.08819
myo-Inositol	1.07972
Isobutyrate	1.01047
Dimethylamine	1.00804

VIP, variable important in projection; MCO, medium cut-off; HF, high flux.

### Proteomic analysis of 1st HF and MCO dialyzer periods

**Figure 3:**
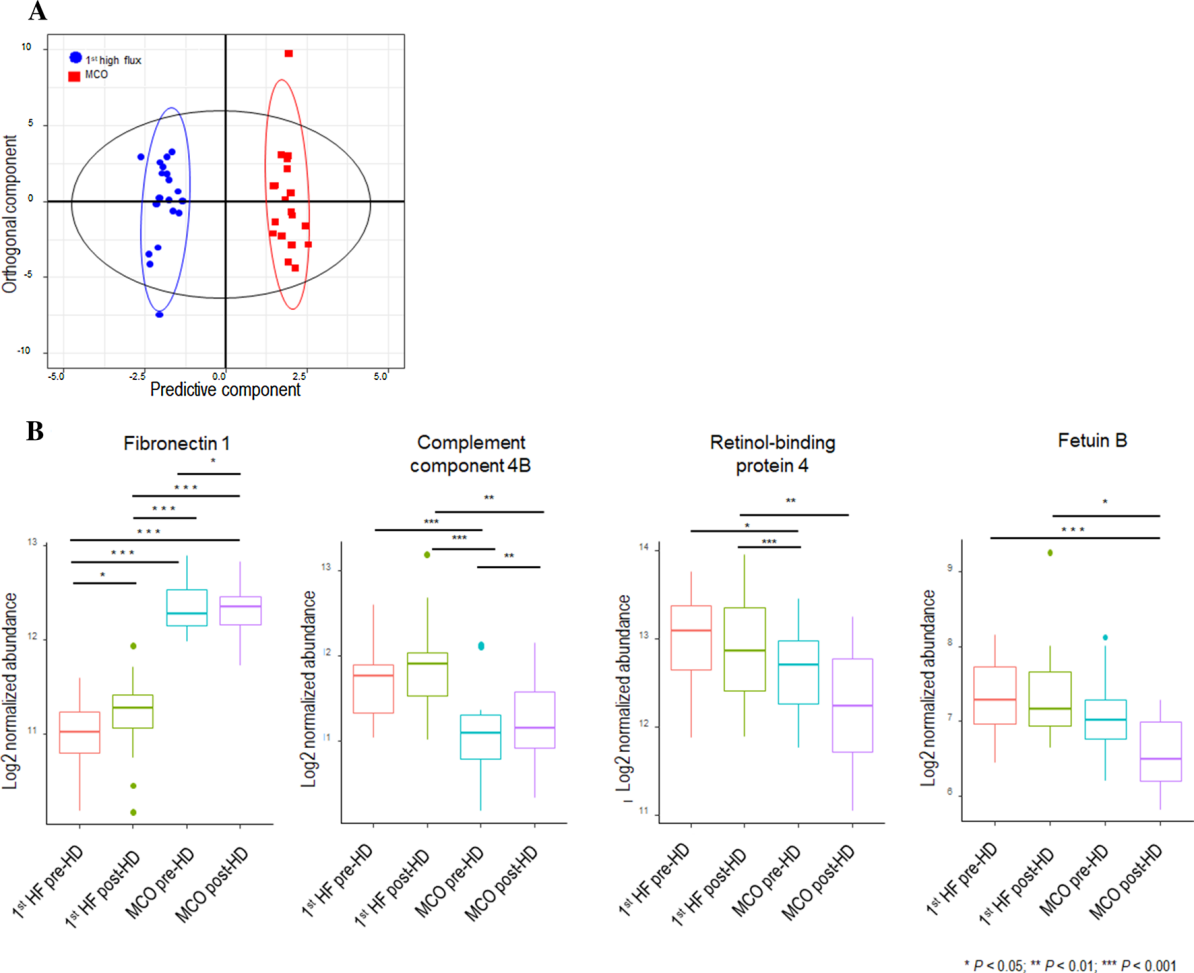
Proteomic differences between the 1st HF and MCO dialyzer periods. (**A**) OPLS-DA score scatter plot derived from the ^1^H NMR spectra of pre-dialysis plasma obtained from subjects in the 1st HF and MCO dialyzer periods. In the OPLS-DA result, the score scatter plot (R^2^ = 0.975, Q^2^ = 0.591) shows a substantial separation of the 1st HF (blue circle) and MCO (red square) dialyzer periods suggesting significant differences between these groups. (**B**) Pre-HD and post-HD normalized protein abundance between 1st HF and MCO dialyzer periods. The *P* values were calculated using the paired t-test using RStudio (version 1.1.456) in R (version 3.6.0): * < 0.05, ** < 0.01, *** < 0.001. The statistical R software packages used included ggplot2 for drawing plots, stats for calculating t-test values, and ropls-package for OPLS-DA for multivariable analysis and feature selection. OPLS-DA, orthogonal partial least-squares discriminant analysis; NMR, nuclear magnetic resonance; HF, high flux; MCO, medium cut-off; VIP, variable important in projection; HF, high flux; HD, hemodialysis.

Using sequential window acquisition of all theoretical mass spectra (SWATH)-mass spectrometry (MS) with multivariable analysis, we determined the plasma protein signatures of patients treated with HF and MCO dialyzers. A total of 666 proteins were quantified from LC–MS/MS analysis of the study samples. However, to minimize the bias of statistical analysis due to missing values, only 127 proteins, detected in all samples were selected for analysis. Therefore, statistical analysis was started with 127 of 666 proteins quantified in all samples without being affected by the MARS14 column. The OPLS-DA score scatter plot (R^2^ = 0.975, Q^2^ = 0.591) showed that the 1st HF and MCO dialyzer conditions were separated substantially from each other, indicating significant differences between these two groups (Fig. 3A). Proteins with a VIP score > 1.0 between 1st HF and MCO dialyzer samples are shown in Table 3; the fold change values are also presented in Table 3. Of note, these fold changes refer to the MCO/1st HF ratio; therefore, values < 1.0 suggest a decrease in protein expression during the MCO period, while values > 1.0 suggest an increased protein expression during the MCO period. Ultimately, among the differentially expressed proteins with significant concentration differences in the MCO period compared to the HF period, we selected four: fibronectin 1 (FN1), the nutritional indicator with the highest VIP score, complement component 4B (C4B), an inflammatory marker, retinol-binding protein 4 (RBP4), a contributor to insulin resistance, and fetuin B (FETUB), a hepatokine that is associated with glucose homeostasis. The pre- and post-HD normalized protein abundances of these markers in the 1st HF and MCO samples are presented in Fig. 3B. FN1 abundance for pre-HD was significantly higher in the MCO period than in the 1st HF period (*p* < 0.001). FN1 abundance was also significantly increased during the single HD session of the 1st HF and MCO dialyzer periods. Conversely, C4B abundance for pre-HD was significantly lower in the MCO period than in the 1st HF period (*p* < 0.001). Similarly, many other complement components (complement component C8 alpha chain, complement component C8 beta chain, complement component 4A, complement factor H, and complement C2) were also significantly decreased (fold change of MCO/1st HF < 1.0) during the MCO period (Supplemental Fig. 1A), as were those of the coagulation factors X and IX (Supplemental Fig. 2B). RBP4 (*p* < 0.05) abundance for pre-HD was significantly lower in the MCO period than in the 1st HF period. Although not statistically significant, FETUB abundance for pre-HD tended to be lower and post-HD FETUB values were significantly lower in the MCO period than in the 1st HF period.

**Table 3:** VIP score of proteins differentially expressed between the 1st HF and MCO dialyzer groups.

Proteins	VIP score	Fold change (MCO/1st HF)*	Proteins	VIP score	Fold change (MCO/1st HF)*
Fibronectin 1	5.119615	2.54	Kallistatin	1.32289	0.90
Complement component 4B	3.029756	0.64	Protein AMBP	1.314166	0.92
Complement component C8 alpha chain	2.335666	0.84	Insulin-like growth factor II	1.294252	0.81
Kininogen-1	1.896474	0.86	Complement C1q subcomponent subunit C	1.292219	1.10
Coagulation factor X	1.866246	0.90	Inter-alpha-trypsin inhibitor heavy chain H2	1.242819	1.10
Clusterin	1.739431	0.88	Coagulation factor IX	1.220091	0.87
Complement component C8 beta chain	1.692606	0.88	Cholinesterase	1.204198	1.09
Alpha-1B-glycoprotein	1.682679	1.09	Complement factor H-related protein 1	1.192683	1.10
Retinol-binding protein 4	1.679422	0.81	Coagulation factor XI	1.192235	1.10
Antithrombin-III	1.550786	0.90	Complement factor H	1.128083	0.87
Cystatin-C	1.509769	0.82	N-acetylmuramoyl-L-alanine amidase	1.116067	0.93
Vitamin K-dependent protein S	1.507964	1.14	Vitronectin	1.098568	1.08
Pigment epithelium-derived factor	1.457402	0.90	Hyaluronan-binding protein 2	1.08575	1.09
Alpha-2-HS-glycoprotein	1.445993	0.89	Cadherin-5	1.037051	0.85
Fetuin-B	1.425672	0.82	Keratin, type I cytoskeletal 9	1.020547	0.90
Complement component 4A	1.395734	0.87	Complement C2	1.008092	0.94
Procollagen C-endopeptidase enhancer 1	1.386906	0.86	Lumican	1.003065	0.88
Inter-alpha-trypsin inhibitor heavy chain H1	1.360755	1.06			

*Protein with a fold change of MCO/1st HF < 1.0 means decreased during the MCO period. In contrast, protein with a fold change of MCO/1st HF > 1.0 means increased during the MCO period.

VIP, variable important in projection; MCO, medium cut-off; HF, high flux.

### Disease and biological function analysis using Ingenuity Pathway Analysis (IPA)

**Figure 4:**
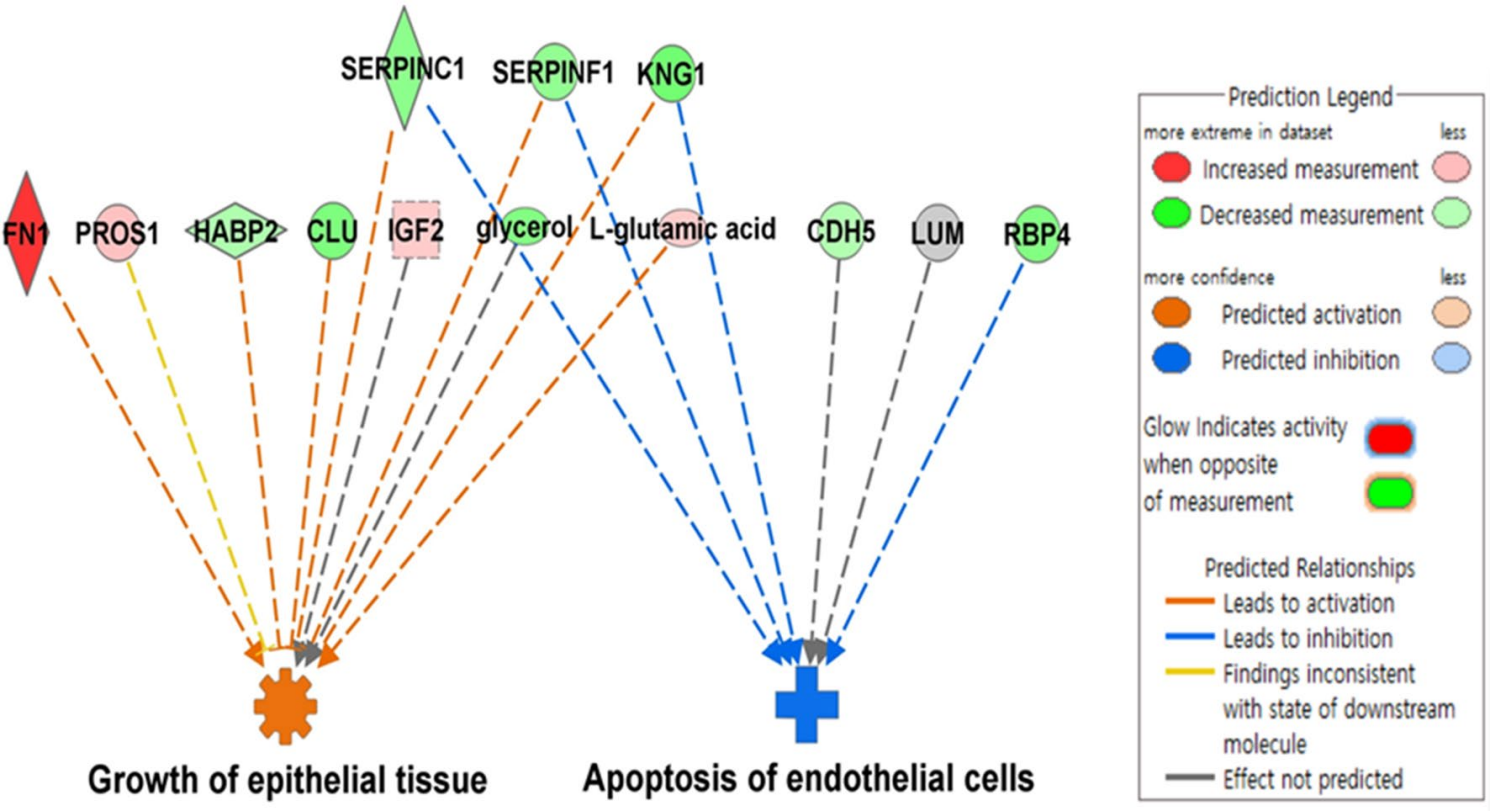
Disease and biological function analysis of the differentially expressed metabolites and proteins using IPA. IPA (QIAGEN Redwood City, CA, USA, http://www.qiagen.com/ingenuity) was used for the integrated analysis of metabolite and protein data. The color of each node (metabolites or proteins) indicates a change in the expression: red (upregulated) and green (downregulated). The edges indicate the predicted relationship between nodes and biological function: orange representing activation, blue representing inhibition, gray representing effect not predicted. The cogwheel and blue cross shapes represent each biological function: orange representing activation, and blue representing inhibition. IPA, ingenuity pathway analysis; GLU, clusterin; FN1, fibronectin 1; HABP2, hyaluronan binding protein 2; IGF2, insulin like growth factor 2; KNG1, kininogen 1; PROS1, protein S; SERPINC1; serpin family C member 1, SERPINF1, serpin family F member 1, CDH5, cadherin 5; LUM, lumican; RBP4, retinol binding protein 4.

Next, we performed an integrated analysis of metabolomics and proteomics using IPA. The 15 significantly changed metabolites, and 35 significantly changed proteins were analyzed. As shown in Table 4 and Fig. 4, 13 of these molecules were significantly enriched in two biological functions: growth of epithelial tissues and apoptosis of endothelial cells (B–H *p*-value = 0.000971 and 0.000375, respectively). Clusterin (*CLU*), *FN1*, glycerol, hyaluronan binding protein 2 (*HABP2*), insulin like growth factor 2 (*IGF2*), kininogen 1 (*KNG1*), l-glutamic acid, protein S (*PROS1*), serpin family C member 1 (*SERPINC1*), and serpin family F member 1 (*SERPINF1*) are involved in the growth of epithelial tissue-related processes, predicted to be activated in MCO compared to levels in the 1stst HF (activation z-score = 1.913). Cadherin 5 (*CDH5*), *KNG1*, lumican (*LUM*), *RBP4*, *SERPINC1*, and *SERPINF1* are involved in the apoptosis of endothelial cells and related processes, and this process was predicted to be inactivated in MCO compared to that in the 1st HF (activation z-score =  − 1.985).

**Table 4:** Disease or function analysis using IPA.

Categories	Diseases or functions	B–H *p*-value^A^	Activation z-score^B^	Molecules
Tissue development	Growth of epithelial tissue	0.000971	1.913	CLU, FN1, glycerol, HABP2, IGF2, KNG1, L-glutamic acid, PROS1, SERPINC1, SERPINF1
Cell death and survival, organismal injury and abnormalities	Apoptosis of endothelial cells	0.000375	− 1.985	CDH5, KNG1, LUM, RBP4, SERPINC1, SERPINF1

Integrated analysis of metabolites and proteins with VIP scores > 1.0 between 1st high flux and MCO dialyzer.

^A^Benjamini–Hochberg (B–H) *p*-value is the adjusted *p*-value using the B–H procedure.

^B^Activation z-score was calculated by IPA software and was used to predict whether a specific disease or biological function was increased (positive z-score) or decreased (negative z-score).

MCO, medium cut-off; IPA, ingenuity pathway analysis; VIP, variable important in projection; CLU, clusterin; FN1, fibronectin 1; HABP2, hyaluronan binding protein 2; IGF2, insulin like growth factor 2; KNG1, kininogen 1; PROS1, protein S; SERPINC1; serpin family C member 1, SERPINF1, serpin family F member 1, CDH5, cadherin 5; LUM, lumican; RBP4, retinol binding protein 4.

## Discussion

Blood metabolism in patients who undergo HD has been reported to change in response to HD. However, dialyzer-dependent changes in metabolites and proteins remain poorly understood. Here, we performed HD using an initial HF approach (1st HF), followed by an MCO approach, and a subsequent second HF approach (2nd HF)—5 weeks each—to examine the metabolite and protein changes over each dialyzer period. The biochemical parameters did not significantly differ among dialyzer periods and albumin levels did not decrease during the MCO dialyzer period. On the other hand, the proteomic and metabolomic profiles, as per the OPLS-DA score scatter plots from the ^1^H NMR spectra and LC–MS/MS results of pre-dialysis samples obtained from subjects in the 1st HF and MCO dialyzer periods showed substantial differences. Pre-HD succinate and histidine were significantly lower, while those of glutamate were significantly higher in the MCO period than in the HF period. Additionally, FN1 abundance in pre-HD was significantly higher and those of C4B and RBP4 were significantly lower in the MCO period compared with 1st HF period. Therefore, overall this study shows that the HD treatment using an MCO dialyzer resulted in different metabolomic and proteomic profiles compared to that using HF dialyzer.

Succinate receptor 1 (SUCNR1) is novel detector of local stress, including hypoxia, ischemia, toxicity, and hyperglycemia^16^. SUCNR1 acts as a sensor of local stress that influences cellular metabolism, as reflected by the increased formation and release of succinate. Local levels of succinate in the kidneys also stimulate the renin-angiotensin system and together with SUCNR1 might play a role in the development of hypertension and the complications associated with metabolic disease and diabetes mellitus^17^. Additionally, in a previous experimental study, succinate was found to have an important role in cardiomyocyte hypertrophy during a heart ischemia event^18^. In the present study, the levels of succinate tended to decrease during the MCO dialyzer period compared to those detected during the 1st HF dialyzer period; interestingly, the levels of succinate were restored during the 2nd HF dialyzer period compared to the MCO dialyzer period. If there is a large reduction in the ultrafiltration volume during HD, intradialytic hypotension can occur^19^, as can hypoxemia, as a consequence^20^. Therefore, hypoxemia can also occur if the reduction in ultrafiltration volume is large, and this can also occur when there is an excessive extracorporeal circulation volume. The Theranova 400 dialyzer has a smaller inner diameter and needs a lower blood compartment volume compared to that with the Polyflux 170 dialyzer. Therefore, the use of the MCO dialyzer might result in less exposure to hypoxic conditions because of low extracorporeal circulation volumes during HD. If succinate level decreases further using the MCO dialyzer, it might decrease the risk of secondary heart damage from HD.

Glutamate is the major nitrogen donor in metabolism and is the most abundant free amino acid in the brain^21,22^. Additionally, it performs excitatory action in the nervous system, which is related to learning and memory. Within the kidneys, glutamate contributes to the secretion of ammonia and the regulation of the acid–base balance^23^. Generally, glutamate requires an appropriate concentration in a certain location in the body, especially in the brain. Therefore, too much or too little glutamate content can be harmful to the human body^22^. Previous studies in humans have demonstrated that high levels of glutamate play a crucial role in inducing nerve damage after brain injury^24,25^. Glutamate is usually higher in dialysis patients than in healthy individuals and glutamate levels have been shown to decrease following HD^14,26^. In the present study, the pre-dialysis glutamate concentrations were higher in the MCO dialyzer period compared to in the 1st HF dialyzer period. Patients in the present study did not have an acute illness or brain injury, and the appropriate concentration of glutamate in HD patients is not well known.

A previous study showed that FN in patients with dialysis is correlated with nutrition markers^27^. FN was found to be positively correlated with the nitrogen balance, serum prealbumin, and transferrin. In addition, the low serum FN levels were elevated after increased protein intake in peritoneal dialysis patients. This is in line with the notion that patients with HD are at high risk for malnutrition and protein-energy wasting, which is, in turn, an independent risk factor for mortality and morbidity^28^. The higher levels of FN1 detected during the MCO dialyzer period in this study might reflect good nutritional status; however, further large-scale long-term studies are needed to prove this hypothesis.

Complement activation occurs during HD, which is related to dialyzer characteristics^29^. Although a biocompatible dialyzer has decreased immunoreactivity, complement activation during HD is still an important issue. This causes the induction of inflammation, promotion of coagulation, and impairment of the host defenses because of the accelerated consumption of complement proteins; ultimately, this can lead to cardiovascular events and mortality^30,31^. In the complement pathway, C4B is in the initiation phase; interestingly, in the present study, levels of many complement components, including C4B, were significantly decreased during the MCO period. Additionally, levels of several coagulation factors also decreased during the MCO period. High C4 levels were associated with the incidence of cardiovascular disease, independently of traditional cardiovascular risk factors in the general population^32^. Additionally, in another study, C4 was found to be associated with CRP; subjects with higher C4 levels showed a significantly higher risk of atrial fibrillation^33^. In addition, dialysis patients exhibit a prothrombic tendency^34,35^ and increased clot structure^36^, which can lead to cardiovascular disease, morbidity, and mortality. Therefore, the reduction in the levels of complement and coagulation factors detected using the MCO dialyzer is expected to help improve the prognosis of HD patients.

RBP4, a 21-kilodalton protein produced by adipocytes, is also chiefly excreted by the kidney and plays a pivotal role in insulin resistance (IR)^37^. In turn, IR is significantly associated with cardiovascular risk, and can significantly predict cardiovascular disease and mortality in dialysis patients^38^. Additionally, RBP4 is also known to be associated with inflammation and oxidative stress^39^. The levels of RBP4 are higher in HD patients than in healthy controls and positively correlate with IR^37^. However, HDF and kidney transplantation both lead to the reduction of the circulating RBP4 levels. Overall, our results, showing a decrease in the RBP4 levels during the MCO period suggest this approach may improve the clinical outcomes of HD patients.

FETUB is secreted by hepatocytes and is upregulated in hepatic steatosis, and impacts impaired glucose metabolism^40,41^. Fetuin A and FETUB are hepatokines that are upregulated in the state of hepatic steatosis. In previous studies, fetuin A was shown to induce IR through inflammatory substances and is, thus, involved in glucose regulation^41,42^. FETUB shares 22% homology with fetuin A. FETUB was also involved in glucose homeostasis; however, contrary to that observed for Fetuin A, the association between IR and inflammation was not significant in FETUB. In this study, the decreased FETUB levels during the MCO period suggest that this HD approach may improve the clinical outcomes of HD patients.

In this study, IPA was also performed to integrate the relationships between differentially expressed metabolites and proteins; this analysis highlighted two main processes: epithelial cell proliferation and endothelial cell apoptosis. The complement activation and inflammation are hallmarks of HD patients, at higher risk of cellular injury and vascular damage^43^. The induction of inflammation is associated with HD per se and the accumulation of cell-activating substances in the blood^44^. HD is also reported to promote apoptosis and lead to the decreased nitric oxide synthesis in cells of the innate and adaptive immune system^31^. This can lead to immune deficiency and endothelial dysfunction in HD patients. Regarding the effect of the MCO dialyzer on plasma proteins during HD, a previous clinical trial showed that it ensures the efficient clearance of pro-inflammatory cytokines^45^. In another study, treatment of renal tubular epithelial cells with the dialysate samples obtained using an MCO dialyzer resulted in a significantly decreased cell viability and the disruption of cell–cell connections, which were confirmed by proteomic analysis compared to the results obtained with dialysate samples obtained using a high flux dialyzer^46^. In addition, the use of an MCO dialyzer enabled more efficient removal of cell-activating and toxic substances^46^. Importantly, the results of our integrated analysis are in line with those abovementioned. Therefore, one can assume that the MCO dialyzer might contribute to the better removal of toxic compounds and improve organ function via an increase in epithelial cell proliferation and the inhibition of endothelial cell apoptosis. These processes are linked to the results described previously herein, such as the decrease in complement activation and inflammation.

This study reports the first comprehensive trial to investigate the difference between two HD dialyzers using two different “omics” approaches. However, there are few limitations of this study. This is a single-center study from Korea; thus, extrapolations of our results to other ethnicities should be approached with caution. This study was conducted as an exploratory study, and it was conducted as a pilot in available patients. Therefore, not many patients were included in the study, which may also be a limitation. However, it is meaningful to see a significant difference in the levels of metabolites and proteins depending on the use of different dialysis membranes, even in a small number of patients. Additionally, several confounders in the context of metabolomic and proteomic analyses cannot be excluded because the proteome and metabolome are also influenced by sex, age, diet, and diurnal variations^10^. For example, some patients were treated with HD in the morning and others were treated in the afternoon, and thus, we could not address the diurnal variation.

In conclusion, our results show that the MCO dialyzer is associated with characteristic metabolomic and proteomic profiles during HD compared to those using the HF dialyzer. These findings might be related to oxidative stress, IR, the complement-coagulation axis, inflammation, and nutrition. A further large-scale study is needed to address the benefits and clinical relevance of the MCO dialyzer for HD patients.

## Methods

### Subjects and study design

This study was designed as a single-center prospective trial on patients undergoing maintenance HD. Twenty patients treated with maintenance HD for more than 3 months at Pusan National University Hospital were enrolled, and the study was conducted from August 2019 to November 2019. This study was approved by the Pusan National University Hospital Institutional Review Board (D-1906-020-079). All clinical investigations were conducted in accordance with the guidelines of the 2008 Declaration of Helsinki. This study was registered with the Clinical Research Information Service (CRIS) at the Korea Centers for Disease Control and Prevention (KCT0004130; registration date: 04/07/2019). The detailed inclusion and exclusion criteria are provided at CRIS (http://cris.nih.go.kr). Briefly, 20 patients receiving maintenance HD for more than 3 months were enrolled. Other inclusion criteria were as follows: (i) older than 19 years of age, (ii) vascular access via arteriovenous fistula or graft, and (iii) with the blood flow rate of more than 250 mL/min. The exclusion criteria were as follows: (i) HD less than three times per week, (ii) HD with peritoneal dialysis, (iii) receiving inpatient treatment for serious disease within the period, (iv) severe fluctuation in blood pressure and intractable fatigue during HD, (v) frequent changes in dry weight and dialysis prescription, (vi) maintenance of dialysis with a temporary catheter, (vii) active malignancy, (viii) enrollment in other clinical trials, and (ix) pregnancy. All patients provided written informed consent.

After enrollment, different dialyzers were used for a 15-week study period as follows: (1) 1st HF dialyzer for 5 weeks, (2) MCO dialyzer for 5 weeks, and (3) 2nd HF dialyzer for 5 weeks to evaluate the short-term maintenance of the MCO efficacy (Fig. 1). The Polyflux 170H (Baxter International Inc., Hechingen, Germany) was used as the HF dialyzer and the Theranova 400 (Baxter International Inc.) as the MCO dialyzer. The detailed characteristics of the dialyzers are presented in Supplemental Table 3. The recommended dialysis prescriptions were as follows: dialysis time, 4 h; blood flow rate, 300 mL/min; dialysate flow rate, 600 mL/min; target single-pool Kt/V > 1.4. To assess the efficacy of the different dialyzers, blood samples were obtained mid-week during the last (the fifth) week of each dialyzer period.

### Clinical data collection and sampling

The baseline demographic and HD-related data including the age, sex, comorbidities, etiology of kidney failure with replacement therapy, and HD duration were recorded. Biochemical data were evaluated during HD (middle of the fifth week of each dialyzer period); the serum biochemical parameters were measured using routine laboratory methods. In our hospital, we used a creatinine method that has calibration traceable to an IDMS reference measurement procedure^47^.

Blood samples were collected before and after the dialysis session in tubes with a serum- or plasma-separating agent and then centrifuged for 15 min at 3,000 rpm and 4 °C. Then, the serum and plasma samples were immediately frozen and stored at − 80 °C until metabolomic or proteomic analyses. The post-dialysis levels were adjusted in consideration of the hemoconcentration, divided by [1 + (intradialytic weight loss [kg]) / (0.2 × end dialysis body weight [kg])]^48^.

### Sample preparation for metabolomics

Before the NMR experiment, frozen serum samples were thawed at room temperature and centrifuged at 15,000 rpm for 15 min at 4 °C. Then, 350 μL of the supernatant was mixed with 350 μL of phosphate buffer solution (0.075 M NaH_2_PO_4_, 0.1% sodium azide, pH = 7.4, 80% H_2_O:20% D_2_O) including containing 4 mM sodium 3-trimethylsilyl-2,2,3,3-d_4_-propionate (TSP-d_4_). The samples were mixed, and after centrifugation (15,000 rpm, 15 min, 4 °C), 600 μL of supernatant was transferred into a 5 mm NMR tube.

### ^1^H-NMR measurements

All NMR spectra were acquired using a 600 MHz Agilent NMR spectrometer (Agilent Technologies, CA, USA). A Carr-Purcell-Meiboom-Gill (CPMG) pulse sequence with pre-saturation with water was used to suppress the macro-molecule and water peak. The ^1^H-NMR spectra were measured using 9.8 µs 90° pulses, an acquisition time of 3.0 s, a total echo time of 64 ms, a relaxation delay of 3.0, 128 scans, and 13 min of total acquisition time.

### ^1^H-NMR spectral pre-processing and serum metabolite identification

Each ^1^H-NMR spectrum was automatically processed through phase and baseline collection, using the Chenomx NMR Suite 8.2 software (Chenomx Inc., AB, Canada). The TSP-d_4_ peak was used for reference to calibrate the chemical shifts. The quantification of the metabolites was accomplished with Chenomx NMR suite 8.2 software. Each peak of the ^1^H-NMR spectrum was assigned and quantified by matching it with the Chenomx NMR suite 600 MHz library database and comparing it to the peak of 2 mM TSP-d_4_ at 0 ppm as the standard peak. For the accurate identification of metabolites, single and overlapping signals were confirmed by spike-in experiments and 2-dimensional correlation spectroscopy (COSY) NMR spectra.

### Sample preparation for proteomics

Plasma samples were sequentially prepared by depletion and digestion steps. First, 40 μL of plasma was injected into the MARS14 column (100 × 4.6 mm; Agilent Technology, Palo Alto, CA, USA) on a binary HPLC system (20A Prominence, Shimadzu, Tokyo, Japan). For this, the mixture was fourfold diluted with proprietary “Buffer A” and loaded onto the MARS14 column on a Shimadzu HPLC system. The unbound fraction was lyophilized with a cold trap (CentriVap Cold Traps, Labconco, Kansas City, MO, USA). Second, protein digestion was conducted using suspension-trapping sample preparation^49^. A 400 µL aliquot of 5% SDS in 50 mM TEAB (pH 7.55) was used for resuspension, and disulfides were reduced by adding dithiothreitol to a final concentration of 20 mM for 10 min at 95 °C. Alkylate cysteines were treated with the addition of iodoacetamide to a final concentration of 40 mM, and samples were incubated in the dark for 30 min after cooling the protein solution at room temperature. The sample was diluted 10 times, and 12% phosphoric acid was added to the sample. The sample was put in S-Trap™ mini columns (ProtiFi, NY, USA, Cat. No: CO2-mini-80), which was performed according to the manufacturer’s protocol, and the ratio of sample to Lys-C/trypsin (Promega Corp., Madison, WI, USA, Cat. No: V5071) was 1:20. The eluted peptide mixtures were lyophilized with a cold trap and stored at − 80 °C until use.

### Mass spectrometry analysis for quantitative proteomics

Before liquid chromatography-tandem mass spectrometry (LC–MS/MS) analysis, dried peptide samples were reconstituted in 0.1% formic acid, and the total peptide concentrations were measured using a UV/Vis spectrophotometer (NanoDrop One, Thermo Fisher Scientific) at a wavelength of 280 nm, with the sample type option set to “1 Abs = 1 mg/mL”. Then, the iRT-standard provided by the iRT-Kit (Biognosys AG, Schlieren, Switzerland) was added into the sample at 1/10 by volume to calibrate the retention time according to vendor instructions^50^. The total amount of each sample was 40 μg, and this was dissolved in 40 μL. The injected 4 μL of sample was analyzed using the SCIEX TripleTOF 5600 + system mass spectrometer with the following LC–MS/MS setup: for LC separation the ekspert nanoLC 425 (Eksigent, Dublin, CA, USA) was used with the Eksigent micro trap cartridge (ChromXP C18CL, 5 μm, 120 Å) as a trap column and the Eksigent column (C18-CL, 0.3 × 150 mm, particle size 3 μm, pore size 120 Å) as an analytical column with the column temperature maintained at 40 °C. The samples were loaded onto a trap column using 100% eluent A:0.1% formic acid at a flow rate of 10 μL/min. After 10 min, peptide mixtures were separated over 57 min using eluent A and eluent B:0.1% formic acid in 100% acetonitrile (eluent B from 3 to 25% over 38 min, 25 to 32% over 5 min, 32 to 80% over 2 min, and kept at 80% for 3 min, 80 to 3% over 1 min and kept at 3% for 8 min) at 5 μL/min. For individual samples, all mass spectrometry runs were operated in the SWATH mode using 100 variable windows, as per the SCIEX technical notes. The SWATH parameters were set as follows: lower m/z limit, 400; upper m/z limit, 1250; window overlap (Da), 1.0; CES was set to 5 for the smaller windows, 8 for the larger windows, and 10 for the largest windows. The MS2 spectra were collected in the range 100–1500 m/z for 2.5 ms in high sensitivity mode, and the total cycle time was 2.8 s. Other MS parameters were set as follows: ion source gas 1 (GS1), 15; ion source gas 2 (GS2), 20; curtain gas (CUR), 30; temperature (TEM), 250 °C; ion spray voltage floating (ISVF), 5500. All MS data have been deposited into the PRIDE archive (www.ebi.ac.uk/pride/archive/) under the Project PXD 026,907 (Pubmed ID: 30,395,289).

### SWATH data analysis

The SWATH-MS data for individual samples were processed using DIA-NN 1.7.10^51^ with a pan-human protein mass spectrometry library^52^. The spectra were searched using the default settings, except *m/z* ranges were 400–1250 for precursors and 100–1500 for fragment ions. Protein quantities were obtained using the MaxLFQ algorithm^53^ as implemented in the DiaNN R package (https://github.com/vdemichev/diann-rpackage); the intensity was filtered for precursors and protein groups that passed the q-value ≤ 0.01 threshold. The protein abundance was normalized as the ratio of the median to the median protein abundance among samples.

### Integrated analysis of the metabolomics and proteomics data

IPA (QIAGEN Redwood City, CA, USA, http://www.qiagen.com/ingenuity) was used for the integrated analysis of metabolite and protein data. The 15 metabolites and 35 proteins selected based on the VIP score > 1.0 threshold were submitted to identify the enriched diseases and biological functions. The *p*-values were calculated using the Fisher’s exact test with a correction for multiple comparisons using the Benjamini–Hochberg method (B–H *p*-value). The activation z-score was calculated to predict whether a specific disease or biological function was activated (positive z-score) or inactivated (negative z-score). A B–H *p*-value < 0.05 and an absolute activation z-score ≥ 2 were considered significant activation.

### Statistical analysis

The baseline clinical characteristics are expressed as the means ± standard deviations or numbers (percentage, %), depending on the nature of the variables. Differences in biochemical parameters among the different dialyzer periods were analyzed using the repeated measures one-way analysis of variance using SPSS, version 20.0 (IBM Co., Armonk, NY, USA). All metabolite concentration values were normalized using the probabilistic quotient normalization (PQN) method and are expressed as the mean ± standard error of the mean. The normalized data were scaled by UV scaling prior to multivariate statistical analysis. To exclude the effects of acetate contained in HD fluids, acetate and the associated metabolites of acetate (3-hydroxybutyrate, acetoacetate, and acetone) were excluded from the analysis. To compare the HF and MCO dialyzer periods, OPLS-DA was performed using SIMCA-P + 12.0 (Umetrics, Umea, Sweden). The quality of the models was defined based on the R^2^ and Q^2^ values; R^2^ is defined as the proportion of variance in the data explained by the models and indicates the goodness of fit, and Q^2^ is defined as the proportion of variance in the data predicted by the model and indicates predictability. The result of OPLS-DA was visualized using a score scatter plot to differentiate between groups. VIP score plots were generated to show the metabolites contributing to the differentiation between each dialyzer period in the OPLS-DA model; VIP > 1.0 was used as the cut-off value for significance. Proteomics data were analyzed using RStudio (version 1.1.456) in R (version 3.6.0). The statistical R software packages used included ggplot2 for drawing plots, stats for calculating t-test values, and ropls-package for OPLS-DA for multivariable analysis and feature selection. The ropls-package for OPLS-DA was used (options are predI = 1, orthoI = NA), and the VIP scores were extracted using the getVipVn function. *p*-values < 0.05 were considered statistically significant.

## Supplementary Information

Supplementary Information.

## Data Availability

All data generated or analyzed during this study are included in this published article, supplementary data, and PRIDE archive.
