# Supplementary material for: Comparative analysis of therapeutic effects between medium cut-off and high flux dialyzers using metabolomics and proteomics: exploratory, prospective study in hemodialysis
Source: Sci Rep. 2021 Aug 30;11:17335. doi: 10.1038/s41598-021-96974-5 (PMC8405670; doi:10.1038/s41598-021-96974-5)
Supplement: Supplementary file 1 — Supplementary Information. [file 41598_2021_96974_MOESM1_ESM.pdf]

Comparative analysis of therapeutic effects between medium cut-off and high flux dialyzers using metabolomics and proteomics: exploratory, prospective study in hemodialysis

Hyo Jin Kim[1,2], Eun Young Seong[1,2], Wonho Lee[3], Suhkmann Kim[3], Hee-Sung Ahn[4], Jeonghun Yeom[5], Kyunggon Kim[4,6], Chae Hwa Kwon[2], Sang Heon Song[1,2]

[1]Department of Internal Medicine, Pusan National University Hospital, Busan, Korea.

[2]Biomedical Research Institute, Pusan National University Hospital, Busan, Korea.

[3]Department of Chemistry, Center for Proteome Biophysics and Chemistry Institute for Functional Materials, Pusan National University, Busan, Korea

[4]Asan Institute for Life Sciences, Asan Medical Center, Seoul, Korea

[5]Convergence Medicine Research Center, Asan Institute for Life Sciences, Seoul, Korea

[6]Department of Biomedical Sciences, University of Ulsan College of Medicine, Ulsan, Korea

Supplemental Table 1. Clinical characteristics of the study population at enrollment

Characteristics		Subjects (N = 20)
Age (years)		59.9 ± 14.2
Sex, male, n (%)		10 (50.0)
DM, n (%)		10 (50.0)
HTN, n (%)		15 (75.0)
Cause of KFRT, n (%)	Diabetic nephropathy, n (%)	5 (25.0)
	Hypertension, n (%)	2 (10.0)
	CGN, n (%)	3 (15.0)
	ADPKD, n (%)	3 (15.0)
	Others, n (%)	7 (35.0)
Hemodialysis duration (months)		92.1 ± 54.6
Vascular access, native (%)		16 (80.0)
Dry weight (kg)		55.4 ± 10.7

DM, diabetes mellitus; HTN, hypertension; KFRT, kidney failure with replacement therapy; CGN. Chronic glomerulonephritis; ADPKD, autosomal dominant polycystic kidney disease

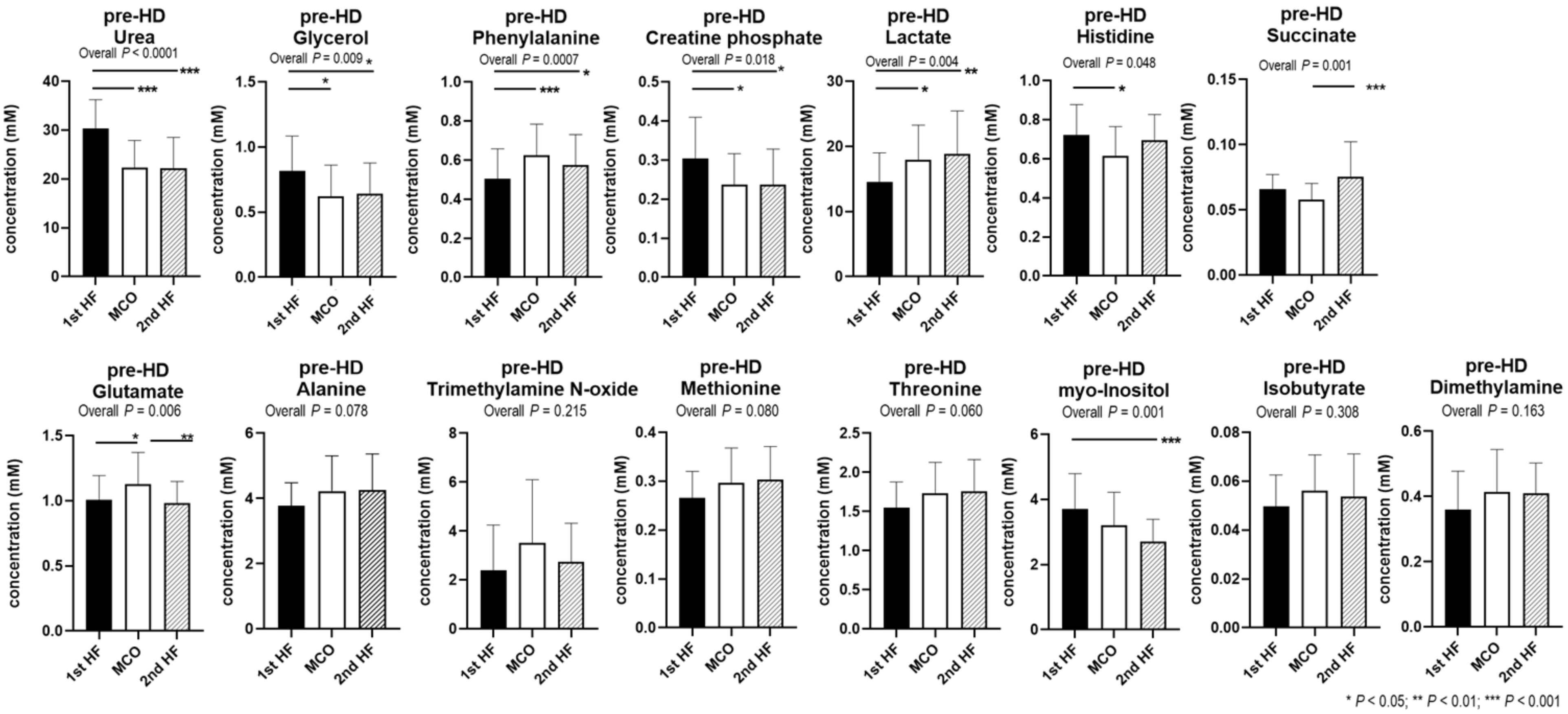

Supplemental Figure 1. Pre-HD concentration of metabolites with a VIP > 1.0 between the 1st HF and MCO dialyzer

The *P* values were calculated using the repeated measures one-way analysis of variance using GraphPad Prism version 9.0.0 for Windows (GraphPad Software, San Diego, California USA, www.graphpad.com): * <0.05, ** < 0.01, *** <0.001.

VIP, variable important in projection; MCO, medium cut-off, HF, high flux

Supplemental Table 2. VIP scores of metabolites differentially expressed between the MCO and 2nd HF dialyzer samples

Var ID (Primary)	VIP Score
Serine	3.37737
Succinate	2.1161
Glutamate	1.8212
myo-Inositol	1.54842
Histidine	1.50569
Asparagine	1.26922
Isoleucine	1.08712
Betaine	1.08627
2-Phenylpropionate	1.07238
Glutamine	1.03373

VIP, variable important in projection; MCO, medium cut-off; HF, high flux

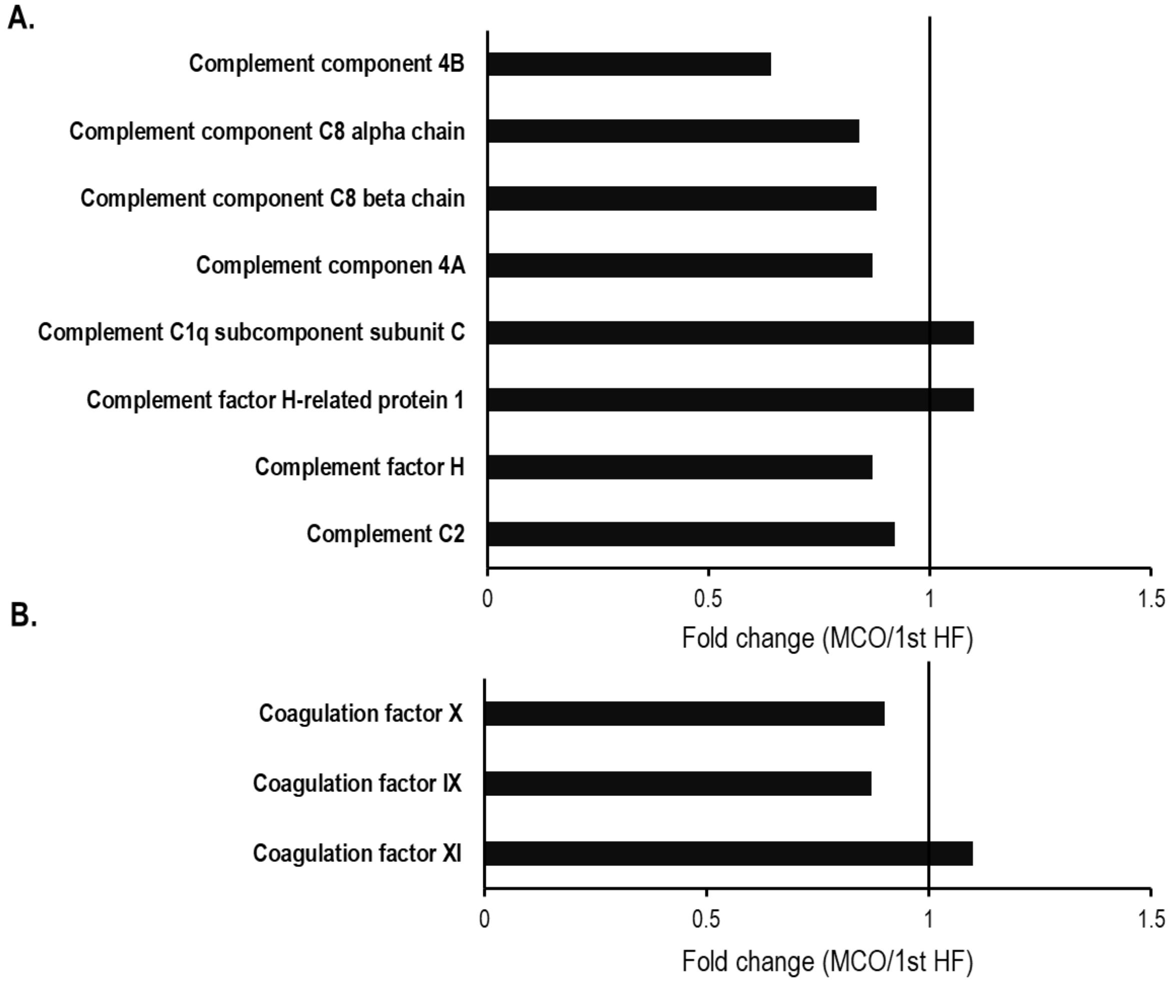

Supplemental Figure 2. Complement components and coagulation factors change between the pre-HD 1st HF and MCO dialyzer samples

A. Differentially expressed proteins of complement components with VIP scores > 1.0 between the pre-HD 1st HF and MCO dialyzer samples

   The vertical line at a fold change of 1.0 represents no changes between the pre-HD 1st HF and MCO dialyzer period. The levels of several complement components, including C4b, were significantly decreased (fold change of MCO/1st HF < 1.0) during the MCO period.

B. Differentially expressed proteins of coagulation factors with VIP scores > 1.0 between the pre-HD 1st HF and MCO dialyzer samples

   The vertical line at a fold change of 1.0 represents no changes between the pre-HD 1st HF and MCO dialyzer period. The levels of the coagulation factors X and IX decreased

(fold change of MCO/1st HF < 1.0) during the MCO period.

Supplemental Table 3. Characteristics of the dialyzers used in this study

Dialyzer	Membrane type	Membrane polymer	Sterilization	Ultrafiltration coefficient (mL/h/mmHg)	KoA urea	Blood compartment volume (mL)	Effective surface area (m^2)	Inner diameter (μm)	Wall thickness (μm)
Polyflux 170	High-flux	Polyarylethersulfone, Polyvinylpyrrolidone and Polyamide	Steam	70	1153	115	1.7	215	50
Theranova 400	Medium cut-off	Polyarylethersulfone and Polvinylpyrrolidone	Steam	48	1482	91	1.7	180	35
